# Improved CRISPR/Cas9 knock-in efficiency via the self-excising cassette (SEC) selection method in *C. elegans*

**DOI:** 10.17912/micropub.biology.000460

**Published:** 2021-09-16

**Authors:** George Huang, Bailey de Jesus, Alex Koh, Sara Blanco, Aubrie Rettmann, Ella DeMott, Melynda Sylvester, Cassie Ren, Carrie Meng, Skye Waterland, Anita Rhodes, Persephone Alicea, Abbey Flynn, Daniel J Dickinson, Ryan Doonan

**Affiliations:** 1 Glow Worms Stream, Freshman Research Initiative, Texas Institute for Discovery Education in Science, College of Natural Sciences, The University of Texas at Austin, Austin TX, USA; 2 Department of Molecular Biosciences, The University of Texas at Austin, Austin TX, USA

## Abstract

Streamlined, selection-based CRISPR knock-in protocols for *C. elegans *were first introduced six years ago (Dickinson *et al*. 2015; Schwartz and Jorgensen 2016). Though these selection-based approaches are powerful, one drawback has been the requirement to inject large numbers of P0 worms (~30-60 per gene target). We have found that a combination of high-purity DNA and a lower concentration of Cas9/sgRNA plasmid dramatically improves efficiency, often resulting in multiple independent CRISPR knock-ins via as few as 10 injected worms, comparable to the efficiency of melted dsDNA templates and purified Cas9 protein (Dokshin *et al*. 2018; Ghanta and Mello 2020).

**Figure 1. Improved CRISPR/Cas9 knock-in efficiency via the self-excising cassette (SEC) selection method in C. elegans:**
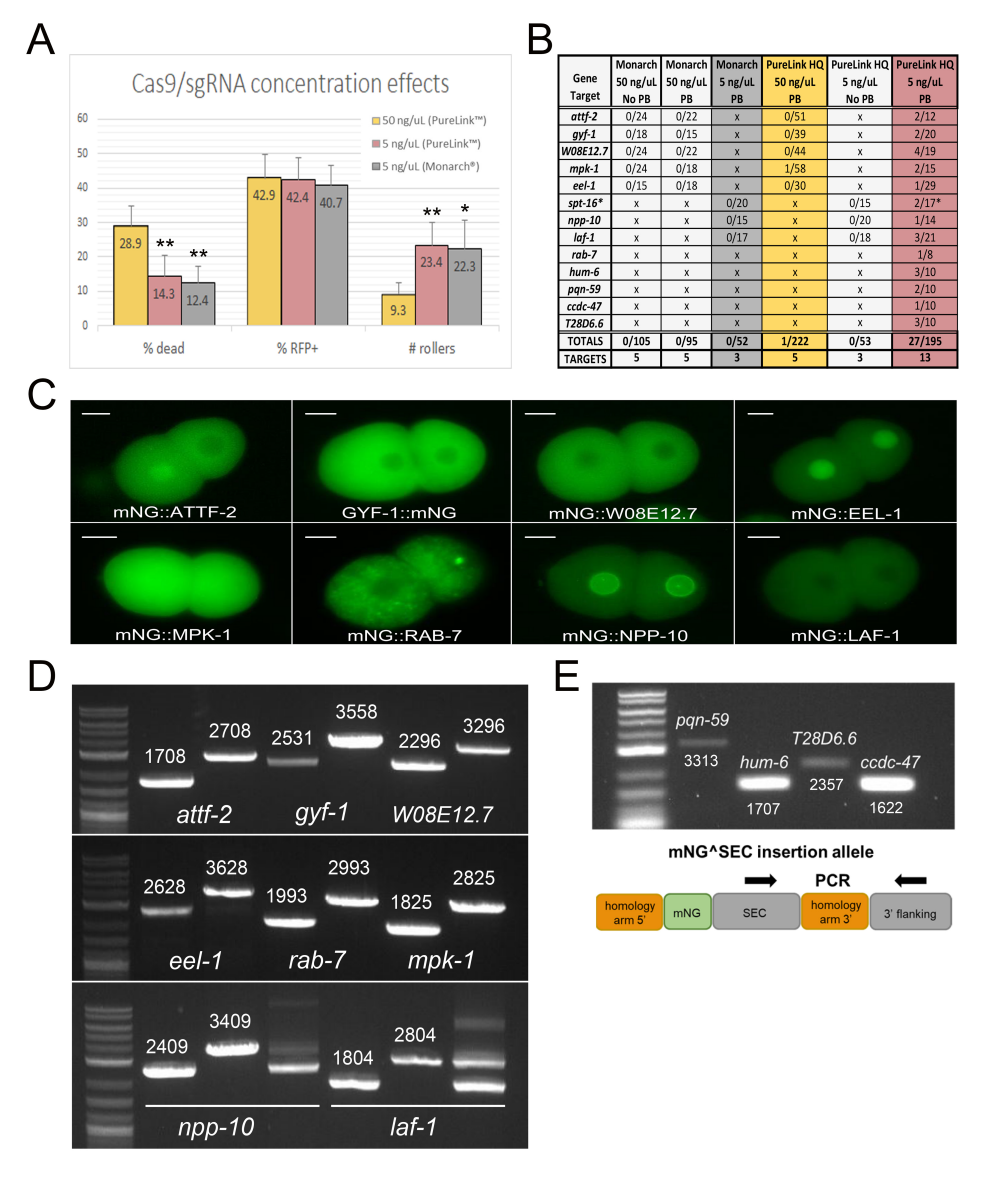
(A) Cas9 concentration affects embryonic viability, whereas DNA quality affects homology-directed repair (but not array formation). P0 worms were injected with either 50 ng/µL or 5 ng/µL Cas9/sgRNA plasmid prepared with a NEB® Monarch® and/or Invitrogen® PureLink™ HQ miniprep kit. 50 ng/µL PureLink™ HQ [n=15 (5 gene targets x 3 P0 injected worms)]; 5 ng/µL PureLink™ HQ [n=24 (8 gene targets x 3 P0 injected worms)]; 5 ng/µL NEB® Monarch® [n=9 (3 gene targets x 3 P0 injected worms)]. *p<0.0001 (2 tailed, unequal variance t-test) **p<0.000001 (2 tailed, equal variance t-test) (B) Lowering Cas9 concentration and using high-quality plasmid DNA dramatically improves fluorescent protein knock-in via CRISPR/Cas9 homology-directed repair. *We have so far been unable to isolate and genotype an N-terminal SPT-16tag due to lethality and balancing issues. (C) Epifluorescence imaging of two-cell stage embryos derived from eight different fluorescent protein knock-in strains. The observed cellular and subcellular expression patterns were consistent with expression data archived in WormBase. Scale bar = 10 microns. (D) Confirmation of knock-in tags via PCR genotyping for the eight strains shown in C. Wild-type amplification (*left*) and tag amplification (*right*) for each gene. *npp-10(utx10)* and *laf-1(utx16)* required balancing to maintain the knock-in allele. PCR genotyping of the balanced heterozygote is shown in addition to genotyping of the wild type and homozygous knock-in. (E) Confirmation of correct insertion site for most recently targeted genes *pqn-59*, *hum-6*, *T28D6.6*, and *ccdc-47*. We have not yet excised these insertions to fully characterize the strains. Primers internal to the SEC region and the 3’ flank of the target gene were used to genotype the insertion site.

## Description

The self-excising cassette (SEC) knock-in approach uses hygromycin selection and a visible roller phenotype to identify knock-ins, followed by a heat-shock induced excision of these visible markers to yield a seamless insertion of a fluorescent protein into the genome (Dickinson *et al.* 2015). Compared to protocols that utilize Cas9 protein and linear DNA repair templates (Paix *et al.* 2015; Dokshin *et al*. 2018; Ghanta and Mello 2020), the plasmid-based SEC approach employs a simpler screening strategy but requires more worms to be injected (Dickinson and Goldstein 2016).

Our goal as a course-based undergraduate research education (CURE) laboratory is to create a collection of fluorescent protein knock-in strains at genetic loci of interest to the research community. Given this focus on making strains, we aimed to determine if the efficiency of CRISPR knock-in via self-excising cassette (SEC) selection could be improved. We were particularly interested in reducing the number of injections required, since injection currently represents the most challenging and labor-intensive step in the procedure.

We first examined embryonic lethality, RFP expression, and rolling in the F1 following injection of individual P0 worms (Figure 1A). Regardless of gene target, we found that injection of the published amount (50 ng/μL) of Cas9/sgRNA plasmid resulted in nearly 30% dead embryos, suggesting that high levels of Cas9 concentration may be toxic. Indeed, an early study using a similar Cas9 expression plasmid found that doses of just 20 ng/μL caused embryonic lethality (Waaijers *et al.* 2013), and a more recent study using purified Cas9 protein found that the number of F1 transgenics was reduced at high Cas9 concentrations (Dokshin *et al*. 2018). Thus, we wondered whether simply reducing the amount of injected Cas9/sgRNA plasmid could improve efficiency. We found that lowering the concentration of Cas9/sgRNA plasmid in the injection mix from 50 ng/μL to 5 ng/μL reduced embryonic lethality by half and increased the number of F1 rollers 2.5-fold (Figure 1A). Interestingly, formation of extrachromosomal arrays was not affected, as ~42% of embryos laid within 16 hours of injection were consistently mCherry+ regardless of Cas9/sgRNA dose or gene target (Figure 1A). Thus, lowering Cas9/sgRNA plasmid concentration allows screening of more F1 transgenics during subsequent selection by reducing Cas9 lethality. Strikingly, this modest 2-fold improvement in embryo survival increased our yield of knock-ins 30-fold, from 0.45% success at 50 ng/μL Cas9 plasmid (one knock-in from 222 P0 injections) to 13.8% at 5 ng/μL (27 independent knock-ins from 195 P0 injections) (Figure 1B). We note that our success rate at 50 ng/μL Cas9 plasmid was lower than previously reported (Dickinson *et al.* 2015), which we attribute to differences in injection technique. Although it is clear from previous reports (Dickinson *et al.* 2015; Heppert *et al.* 2018) that skilled injectors can reliably generate knock-ins using 50 ng/μL Cas9 plasmid, our observations suggest that a reduced Cas9 concentration can facilitate generating knock-ins for non-expert injectors.

In addition, we found that the purity of the injected plasmids was critical for success. We compared NEB® Monarch® versus Invitrogen® PureLink™ HQ plasmid minipreps, with and without an additional Qiagen® Buffer PB (or “homemade” 4M guanidine hydrochloride/30% isopropanol) wash included in the protocol. We were never able to isolate knock-ins with Monarch® preps, even with a Buffer PB wash included and a low Cas9/sgRNA plasmid concentration (Figure 1B). In contrast, injections with Invitrogen® PureLink™ HQ preps yielded substantially better results, but only if a Buffer PB wash step was included in the purification protocol. We determined that this adjustment is critical for success by injecting identical knock-in constructs prepared with and without this wash step (Figure 1B). Overall, in combination with injection of Cas9/sgRNA plasmid at 5 ng/μL, these simple adjustments resulted in an average of 1.5 knock-ins for every 10 F0 worms injected, reaching 2.1 knock-ins for every 10 F0 worms when screening only the worms with a confident injection in both gonad arms (Figure 1B). Importantly, this level of efficiency is now comparable to methods employing direct injection of Cas9-RNP and melted dsDNA repair templates (Ghanta and Mello 2020), but with the added advantages that SEC selection facilitates strain isolation and eliminates the need for PCR-based screening.

An unexpected finding was that Monarch® and PureLink™ HQ preps appeared to show equivalent levels of extrachromosomal array formation, but not knock-in (Figures 1A, B). However, we noticed that F1 array animals derived from PureLink™ HQ injections appeared healthy and were frequently resistant to hygromycin, whereas F1 arrays derived from Monarch® injections were mostly sick and/or had low fecundity when challenged with hygromycin, despite being RFP+ rollers. These anecdotal observations suggest that contaminants present in the Monarch® plasmid preparation interfere with the selection procedure. Overall, we conclude that high-quality plasmid DNA promotes more efficient selection, via a mechanism that is not yet fully understood.

To demonstrate that our improvements to the SEC protocol were consistent across multiple targets, we generated novel mNeonGreen (mNG) knock-in strains for 13 different target genes (Figure 1B). We verified correct mNG insertion for 12 of the 13 gene targets via PCR genotyping (Figure 1D-E). The remaining target, *spt-16,* yielded putative knock-ins based on plate phenotype, but we were unable to maintain the insertion due to lethality. We examined mNG fluorescence for 8 strains and found that, consistent with expression data curated in WormBase, all of the relevant proteins were expressed during early embryogenesis in the correct subcellular compartment (Figure 1C). Although most knock-in strains were healthy, some exhibited unintended phenotypes. mNG::NPP-10 and mNG::LAF-1 required balancing due to sterility and larval lethality, respectively (Figure 1D). mNG::EEL-1 homozygotes were slow growing with a complex pleiotropic phenotype; some animals were superficially wild type, whereas others were Sma, Egl, or embryonic lethal. Thus, insertion of mNG at some of these loci compromised gene function.

Together, these data show that, despite recent advances in gene tagging via Cas9-RNP injection (Dokshin *et al*. 2018; Ghanta and Mello 2020), SEC selection can be equally efficient. We now routinely inject just 10 worms per target per session, which typically results in at least one correct insertion per target (Figure 1B). Selection-based protocols eliminate the need for careful timing and laborious worm picking required in PCR- or fluorescence-based screening approaches, making endogenous gene tagging possible even for novice undergraduate students attempting CRISPR/Cas9 methodology for the first time.

## Methods

*Plasmid preparation*

Cas9/sgRNA and mNG^SEC^3xFlag repair plasmids were designed and created for each gene target as previously described (Dickinson *et al*. 2015). The Invitrogen® PureLink™ HQ minprep (K2100-01) protocol was done exactly as indicated, except for the addition of a 500 µL wash with Qiagen® Buffer PB (Cat # 19066) immediately after binding of the supernatant plasmid DNA to the column. The NEB® Monarch® miniprep (T1010L) was done exactly as indicated or the “Wash 1” step was replaced by a 500 µL wash with Qiagen® Buffer PB.

*CRISPR injection mix*

The CRISPR injection mix was prepared in ultrapure H_2_O at these final concentrations:

· Cas9/sgRNA plasmid: 50 ng/µL or 5 ng/µL

· mNG^SEC^3xFlag repair plasmid: 25 ng/µL

· Co-injection markers: pCFJ90 (2.5 ng/µL) and pCFJ104 (5 ng/µL)

*Microinjection*

All microinjections were done by the same person for consistency (n=759 total worms injected in this study). Injected worms were transferred to NGM plates in pairs or threes for subsequent hygromycin screening or individually for subsequent F1 phenotypic scoring (see below). All P0 worms were scored for surviving injection at 24 hours, and 722 worms survived (i.e. 95%).

*Phenotypic scoring*

Injected worms were cultured individually for 16 hours at 25°C and then transferred to a new NGM plate at 25°C to continue laying eggs. Embryos and larvae of the initial (0-16 hours) F1 were counted and scored for RFP+ at 16 hours and embryonic lethality was scored at 24 hours. Total number of rollers was scored prior to hygromycin treatment 3 days post-injection. Hygromycin resistance was scored by transferring F1 rollers to hygromycin plates as soon as the roller phenotype was observed. Only rollers that reached reproductive maturity and laid eggs were considered hygromycin resistant.

*PCR genotyping*

Single worm lysis and PCR was used to genotype insertion and/or tag alleles for all gene targets using Q5 polymerase via NEB®’s standard Q5 polymerase protocol. All primer sequences available by request.

*gRNA sequences*

**Table:** 

*attf-2*	CGAAACTGCAACCATACCCG
*gyf-1*	TGACTCATCTAACGGCGCGA
*W08E12.7*	GAAAGCAGCTGCTCCAGCGA
*mpk-1*	TTCTTCTTGCAGATGGCCGA
*eel-1*	GACCCGGAAGAGCTTGAACT
*spt-16*	GGGAGAAATGTCTGGAAAAC
*npp-10*	TTCGGCCAGAACAAATCATT
*laf-1*	GAAAGTAACCAATCGAACAA
*rab-7*	CTTCCAGTGAACAAAAATGT
*hum-6*	AACAATGGTATTAGTAAGCA
*pqn-59*	TGCAAGTCGCGCTTGATCGC
*ccdc-47*	CGTTCACCATGAAAATCGTC
*T28D6.6*	GGGAATTTCAGCTTAGACTC

## Reagents

**Table:** 

**Strain**	**Genotype**	**Available from**
GLW2	*attf-2(utx2[mNG::attf-2)] V*	CGC
GLW4	*gyf-1(utx4[gyf-1::mNG]) II*	CGC
GLW6	*W08E12.7(utx6[mNG::W08E12.7]) IV*	CGC
GLW8	*eel-1(utx8[mNG::eel-1]) IV*	CGC
GLW10	*npp-10(utx10[mNG::npp-10])/sC1 III*	Glow Worms lab
GLW16	*rab-7(utx12[mNG::rab-7]) II*	CGC
GLW19	*mpk-1(utx14[mNG::mpk-1]) III*	CGC
GLW21	*laf-1(utx16[mNG::laf-1])/qC1R III*	Glow Worms lab
